# Hints to Practitioners

**Published:** 1898-01-22

**Authors:** 


### HINTS TO PRACTITIONERS.

### Boric Acid Lotion.

For cases of extensive tinea tonsurans.

℞. Acidi Borici, ℥i. vel. q. s.

Ætheris Methylati, ℥x.

Olei Rosmarini, ʒii.

Spiritum methylatum, ad. ℥xl.

Misce. Fiat solutio saturata limpida.

This lotion has to be made up in large quantities at a time, as it must be very freely used; hence it is very much cheaper to employ methylated ether and spirit, and the oil of rosemary is useful to counteract its disagreeable smell.—"*Ringworm and Alopecia Areata,*" *by Dr. Aldersmith.*

### The Inunction of Mercury.

A method of inunction which is recommended by Mr. Swinford Edwards is as follows: Ordinary blue ointment is employed, and the patient is told to put a piece the size of a ten-grain pill on the sole of the foot, rubbing it in after a warm bath for a minute or two, and then drawing on undyed socks, thus walking the mercury in.

### The Morning Cough of Phthisis.

The morning cough of phthisis is a considerable trial great as may be its uses in removing the accumulations of the night. The worst of it is that it is not only exhausting, but too often brings back the breakfast. If the sputa be tenacious, Dr. Graham Crookshank says that an alkaline draught, taken in warm water on first waking in the morning, will be found to loosen the secretion and prevent retching. He suggests the following:—

| | |
|---|---|
| Sodii bicarb. ... ... ... | gr. 10 |
| Sodii chlor. ... ... ... | gr. 3 |
| Ether chlor. ... ... ... | ♏ 5 |
| Aq. anisi ... ... ... ... | fl. oz. I |

We can fully endorse this, and may add that we have often found patients much relieved, and their paroxysms of coughing much shortened, by slowly sipping hot salt and water—a salt cellar spoonful of salt in a teacupful of hot water—flavoured by the addition of a little Liebig's extract of meat.

